# Age of Greatest Susceptibility to Childhood Lead Exposure: A New Statistical Approach

**DOI:** 10.1289/ehp.0800426

**Published:** 2009-05-07

**Authors:** Richard W. Hornung, Bruce P. Lanphear, Kim N. Dietrich

**Affiliations:** 1 Cincinnati Children’s Environmental Health Center, Cincinnati Children’s Hospital Medical Center, Cincinnati, Ohio, USA; 2 Department of Environmental Health, University of Cincinnati College of Medicine, Cincinnati, Ohio, USA; 3 Faculty of Health Sciences, Simon Fraser University and Child and Family Research Institute, British Columbia Children’s Hospital, Vancouver, British Columbia, Canada

**Keywords:** age effects, blood lead, collinearity, temporal pattern

## Abstract

**Background:**

Susceptibility to lead toxicity is often assumed to be greatest during early childhood (e.g., 2 years of age), but recent studies suggest that blood lead concentrations (BPb) taken at 5–7 years of age are more strongly associated with IQ.

**Objective:**

We aimed to determine the age of greatest susceptibility to lead exposure using an innovative statistical approach that avoids the problem of correlated serial BPb measurements.

**Methods:**

We analyzed two cohorts of children that were followed from infancy to 6 years of age in Rochester, New York (*n* = 211), and Cincinnati, Ohio (*n* = 251). Serial BPb levels were measured and IQ tests were done when children were 6 years of age. After adjustment for relevant covariates, the ratio of 6-year BPb to 2-year BPb was added to the multiple regression model to test whether the pattern of BPb profiles during childhood had additional effect on IQ.

**Results:**

The ratio of BPb at 6 years to the BPb at 2 years showed a strong effect on IQ (*p* < .001) when added to the multiple regression model that included the average childhood BPb. IQ decreased by 7.0 points for children whose BPb at 6 years of age was 50% greater than that at 2 years compared with children whose 6-year BPb was 50% less than their 2-year BPb. Similarly, criminal arrest rates were a factor of 3.35 higher for those subjects whose 6-year BPb was 50% higher than their 2-year BPb.

**Conclusions:**

We conclude that 6-year BPb is more strongly associated with cognitive and behavioral development than is BPb measured in early childhood.

Because the U.S. Centers for Disease Control and Prevention (CDC) lowered its level of concern for childhood blood lead concentration (BPb) from 25 to 10 μg/dL in 1991 (CDC 1991), several individual cohort studies and meta-analyses have focused on cognitive developmental effects (psychometric intelligence or IQ) of BPb concentrations < 10 μg/dL. Two meta-analyses that included both cross-sectional and longitudinal studies concluded that there was no evidence of a threshold at 10 μg/dL for effects on childhood IQ, but they did not examine the effects among children who had a maximum BPb concentration < 10 μg/dL (Pocock et al. 1994; Schwartz 1994). An analysis of the Rochester Longitudinal Study (Canfield et al. 2003) reported a decrement of 7.4 points in IQ at 5 years of age associated with an increase in mean childhood BPb levels from 1 to 10 μg/dL for children who had a peak BPb concentration that never exceeded 10 μg/dL. A more recent update of the Rochester cohort (Jusko et al. 2008) found that 6-year-old subjects with a mean childhood BPb concentration < 5μg/dL scored 4.9 IQ points higher than children with mean childhood BPb between 5 and 9.9 μg/dL. An updated analysis of the Boston cohort found similar results among children with maximum BPb levels < 10 μg/dL (Bellinger and Needleman 2003). Although the weight of evidence suggests that adverse effects on cognitive development are associated with BPb concentrations < 10 μg/dL, the age of greatest vulnerability has not been clearly indicated.

In a pooled analysis of seven cohorts, Lanphear et al. (2005) examined four different BPb metrics in relationship to child IQ: mean BPb levels from 6 months to the age closest to the IQ test, early childhood mean BPb levels from 6 to 24 months of age, peak BPb levels measured anytime before the IQ test, and concurrent BPb defined as BPb levels measured closest to the IQ test. That study found the measure of concurrent BPb levels to have the strongest relationship to IQ, but early childhood average, lifetime childhood average, and peak BPb levels were all significantly and inversely related to IQ. No attempt was made in that study to determine the age of greatest association with IQ across the spectrum of all annual BPb measurements. In an analysis of data from a clinical trial of succimer treatment in children with BPb levels between 20 and 44 μg/dL, Chen et al. (2005) attempted to determine whether BPb levels measured at early or late childhood were more strongly related to IQ scores. Their approach was to include both prior and concurrent BPb levels in each model of IQ scores. They concluded that BPb levels measured at 7 years of age was more strongly related to IQ measured at 7 years than the higher BPb levels measured at 2 years of age. That analysis had two important limitations: First, as the authors noted, analysis of both prior and concurrent BPb concentrations in the same model introduced the problem of collinearity between these two measures. Second, all children in this study had high BPb levels by contemporary standards (20–44 μg/dL), thus making generalization to a population with more typical levels somewhat problematic.

A study of the effects of lead on IQ by Wasserman et al. (2000) also addressed the problem of serial correlation of BPb measurements. They examined postnatal BPb relative to prenatal lead exposure in a Yugoslavian cohort of lead-exposed children. To reduce problems with tracking of prospective measurements, they created a categorical variable that characterized early and late postnatal measurements by whether they increased or decreased in relation to prenatal BPb levels. After adjustment for social factors and prenatal BPb, they found that early childhood BPb levels were most strongly related to decrements in IQ, but elevated BPb levels in late childhood were also associated with IQ decrements.

Although recent research has provided a new understanding of the adverse effects of childhood lead exposure at BPb levels < 10 μg/dL, the age of greatest susceptibility is still in question. Recent studies (Chen et al. 2005; Lanphear et al. 2005) suggest that lead exposure beyond 2 years of age, when BPb levels generally peak, may be more strongly associated with cognitive development. However, the strong correlation, or tracking, of BPb levels from infancy to school age makes the determination of the age of greatest susceptibility a difficult task. Brubaker (2008) examined the association of serial BPb measures and reduction in gray matter from magnetic resonance brain imaging of 157 young adults in the Cincinnati Lead Study and found that BPb concentrations at 5 and 6 years of age were more highly associated with gray matter volume reductions in the prefrontal cortex than were BPb levels measured at earlier ages. They made no attempt to examine the effects of multiple BPb measures in the same model, so that analysis also does not directly address the problem of separating early and late childhood lead exposures.

To determine the age of greatest susceptibility, we introduce a new statistical approach to examine the relationship between temporal patterns in childhood BPb levels and subsequent cognitive and behavioral development. Our objective is to estimate the age most highly related to decrements in IQ while reducing or eliminating the bias that may be associated with strong correlation of serial BPb measurements.

## Methods

To address the issue of age of greatest susceptibility, it is necessary to use data from studies that measured BPb serially from infancy through at least 6 years of age. Our primary analysis uses data from the Cincinnati Lead Study and the Rochester Longitudinal Study. The Cincinnati study contained data on 251 children enrolled during 1979–1984 (Dietrich et al. 1993). The Wechsler Intelligence Scale for Children–Revised (Wechsler 1989) IQ test was administered to these children at approximately 6 years of age. The Rochester study included 211 children enrolled during 1994–1995 (Lanphear et al. 1999). The Wechsler Preschool and Primary Scale of Intelligence–Revised (Wechsler 1974) IQ test was administered to these children at approximately 6 years of age. The combined cohort contained 462 children whose BPb concentrations were measured at least annually from infancy to 6 years of age. In both studies, the IQ measured at approximately 6 years of age is considered more stable than IQ tests at earlier ages, and also follows the strategy used in the international pooled analysis (Lanphear et al. 2005).

### BPb concentration

In addition to yearly BPb measurements made annually from 1 through 6 years of age, other BPb metrics were also available. We calculated the childhood average as the mean of all BPb measurements from 6 months through 6 years of age. The peak childhood BPb was defined as the maximum of all BPb measurements during the same period.

### Statistical analysis

We used multiple regression models to investigate the relationship between BPb and IQ scores measured at 6 years of age. In the analysis of the Cincinnati and Rochester cohorts, we included the five covariates identified in the international pooled analysis (Lanphear et al. 2005): site, Home Observation for Measurement of the Environment (HOME) score, birth weight, maternal IQ, and maternal education. The HOME score is an index reflecting the quality and quantity of emotional and cognitive stimulation in the home environment (Bradley and Caldwell 1979). We defined maternal education as the highest grade completed by the subject’s mother.

Our new approach was to examine the temporal pattern of the available longitudinal BPb data while controlling for the overall exposure to lead throughout childhood. Although children’s BPb levels generally peak at approximately 2 years of age and then slowly decline, this is not universally true. This would be the usual scenario if the child’s lead exposure environment was relatively stable from infancy to 6 years of age, primarily due to mouthing behavior. However, the temporal pattern of lead contamination in a child’s environment may change during childhood because of a move by the child’s family to a new location or because of home renovation. The newly occupied or renovated house could contain higher lead contamination that might result in a child’s BPb peaking beyond 2 years of age.

To address the impact of changes in the usual temporal pattern of childhood BPb, we created a new exposure variable defined as the ratio of the BPb level at 6 years of age to the BPb level at 2 years of age. Because a high BPb concentration at 6 years could simply reflect an even higher BPb level at 2 years, we added the log of the childhood average BPb (6 months to 6 years) to the model in order to compare the effects of a range of ratios of 6-year to 2-year BPb levels while holding the overall childhood lead exposure constant. Our objective was to determine whether early or late childhood lead exposure was more detrimental to cognitive development while avoiding the collinearity problem associated with previous analyses containing multiple BPb measures. We assessed the linearity of the relationship of IQ to the ratio by testing the statistical significance of quadratic and cubic terms in the ratio. We also tested whether the ratio effect could be considered constant across the full range of childhood average BPb levels by testing the interaction of the ratio with the childhood average BPb levels. After developing final multiple regression models, we examined regression diagnostics to determine whether collinearity or influential observations could have caused bias in our results (Belsley et al. 1980). We also tested the residuals of the final model to determine whether they followed a normal distribution and then plotted the residuals against the predicted values to determine whether they were homogeneous across the range of predicted values.

We also wanted to determine whether our ratio approach was valid when examining behavioral outcomes other than cognitive development. Accordingly, in addition to examining age effects on IQ scores, we also analyzed the temporal pattern in lead exposure related to adult criminal behavior. A recent analysis of the Cincinnati Lead Study cohort found a statistically significant positive relationship between adult criminal arrest rates and 6-year BPb levels (Wright et al. 2008). In a manner similar to that described for IQ scores, we used the same covariates identified in the model of adult criminal behavior, while adding the childhood average BPb and the 6-year:2-year BPb ratio to that model. Our objective was to examine the effect of variation in temporal patterns of BPb levels on a behavioral outcome other than IQ.

## Results

**Figure 1:**
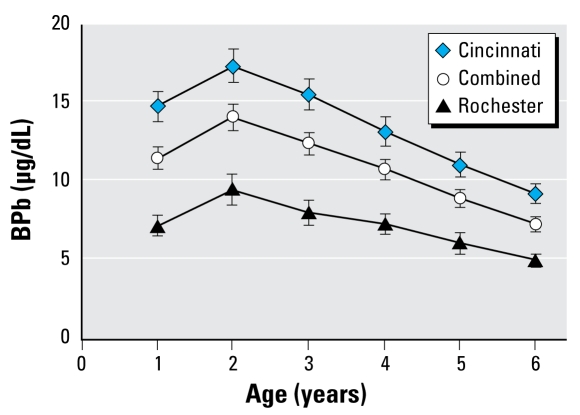
Annual BPb levels and 95% confidence intervals for Cincinnati and Rochester cohorts, individually and combined.

Of the 462 children in the Cincinnati and Rochester cohorts, complete data on BPb levels and covariates were available for 397 (86%) children. We found no significant mean differences for IQ scores or covariates between the 397 included children and the 65 children with one or more missing covariates. Characteristics of each cohort are similar, except for BPb levels and the proportion of nonwhite children (Table 1). The Cincinnati children had mean childhood average BPb concentrations that were approximately twice as high as those of the Rochester children (11.7 vs. 5.8 μg/dL) primarily because they enrolled 12–15 years earlier when lead exposures were more prevalent. The Cincinnati cohort also had a somewhat higher proportion of nonwhite children (89.1 vs. 71.0%). In each of the cohorts, as well as the combined data, the mean BPb levels were highest at 2 years of age (14.1 μg/dL, combined data) and lowest at 6 years of age (7.3 μg/dL) (Figure 1). Childhood average BPb from 6 months to 6 years of age in the combined cohorts was 9.9 μg/dL, with 58% of the children having childhood average BPb < 10 μg/dL. The mean of the ratio of 6-year to 2-year BPb concentrations was 0.58, with a range of 0.11–4.09. There were 25 children (6.3%) with a ratio > 1.0, meaning that the 6-year BPb was greater than the 2-year BPb. The average IQ for children at 6 years of age was 85.9.

Our first step was to calculate the correlation matrix for the log of annual BPb measurements at 1–6 years of age. Table 2 clearly indicates the high degree of serial correlation among these measurements from the combined Cincinnati and Rochester cohorts. This high degree of collinearity precluded us from fitting a model with all six yearly BPb levels in the same model. Instead, we produced six separate models, one for each of the six annual BPb measurements. Table 3 shows the results of these six models. Similar to the results of Brubaker (2008), the estimated coefficients for a log-linear relationship clearly indicate that BPb concentrations at later ages are more strongly related to IQ on a cross-sectional basis. We then specified a model containing terms for the log of mean childhood BPb from 6 months to 6 years of age and the ratio of 6-year to 2-year BPb levels, as well as the five covariates identified above. Because the analysis of BPb at individual years indicated that 5-year BPb was also strongly related to IQ, we created an additional model to examine the 5-year to 2-year ratio.

We examined the effect of variation in the usual pattern of BPb peaking at approximately 2 years of age. The parameter estimates for the 6-year:2-year BPb ratio showed a statistically significant negative effect of the ratio (β = −7.00; 95% confidence interval, −10.00 to −3.99; *p* < 0.001) on IQ after controlling for the average lead exposure during childhood as well as covariates (Table 4). We found no departure from linearity in the ratio effect and no significant interaction of the ratio with childhood average BPb (*p* = 0.28). In a secondary analysis, we also examined the ratio effect after controlling for 6-year BPb instead of average childhood exposure. Results were very similar (β = −6.3, *p* < 0.001), and all subsequent analyses were adjusted for childhood average exposure. Table 5 shows the final model, including all covariates.

**Figure 2:**
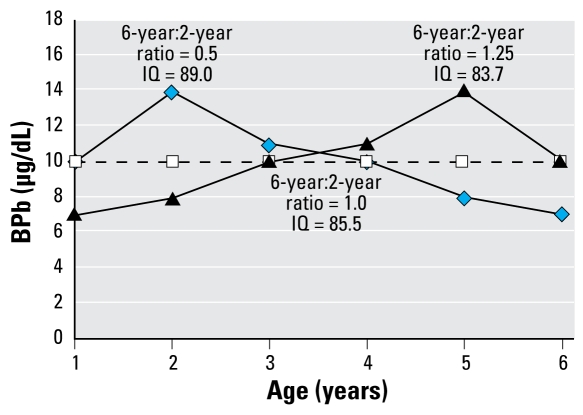
Estimated IQ in combined Cincinnati and Rochester cohorts for three patterns of BPb levels from 1 through 6 years of age: peak at 2 years (diamonds), peak at 5 years (triangles), and constant BPb level (squares). All three scenarios have identical mean BPb level of 10 μg/dL.

This result indicated that for two groups of children with the same average childhood lead exposure, those children who had relatively higher BPb concentrations at 6 years of age had greater decrements in IQ measured at age 6. Figure 2 illustrates this effect for three exposure scenarios: one characterizing the typical pattern with BPb twice as great at 2 years of age as at 6 years of age, another with BPb levels 25% greater at age 6 than at age 2, and a third with BPb constant at the average childhood mean for the first two scenarios. Even though their average BPb from 1 to 6 years of age was identical, the child whose BPb was higher at age 6 had an estimated 5.3 point greater loss in IQ compared with the child whose BPb peaked at age 2. Results were similar when we examined the ratio of BPb levels at age 5 to those at age 2. However, analyses of ratios involving BPb levels at 3 and 4 years of age showed no significant effects (data not shown). Regression diagnostics indicated no problems with collinearity or influential observations in these models. To address internal validity, we also repeated the analyses separately for the Cincinnati and Rochester cohorts. For each individual cohort, the 6-year:2-year ratio was significantly associated with IQ after controlling for average childhood BPb and covariates. In a secondary analysis following our approach in the international pooled analysis (Lanphear et al. 2005), we fit the same model to data from children in the Cincinnati and Rochester cohorts whose maximum BPb level never exceeded 10 μg/dL. Similar to our results in the pooled analysis, the effect of the ratio was even stronger in terms of a larger estimated decrement in IQ for this analysis restricted to less exposed children (β = −12.5, *p* = 0.002). Thus, this statistical approach is applicable to populations with BPb levels in the range above, as well as below, the current CDC “action level” (CDC 1991, 2002).

### Adult criminal arrests

To extend this analysis beyond standardized measures of psychometric intelligence, we next examined the relationship of temporal patterns of lead exposure during childhood to adult criminal arrest rates. As previously described (Wright et al. 2008), we used negative binomial regression to analyze arrest rates from 19 to 24 years of age among 250 members of the Cincinnati Lead Study. The results reported by Wright et al. indicated a statistically significant relationship between arrest rates and prenatal maternal BPb and 6-year BPb concentrations. Following the same analytic strategy that we used in the combined Cincinnati and Rochester study of IQ, our model included mean childhood BPb and the 6-year:2-year BPb ratio plus covariates used in the model developed by Wright et al. (2008): maternal IQ, sex, socioeconomic status (Hollingshead score) (Cirino et al. 2002), and maternal education. Results were similar to those that we found in the IQ analysis (Table 6). The ratios of 6-year and 5-year BPb level to the 2-year BPb level were both significantly and positively associated with the adult arrest rate after controlling for the mean childhood average BPb level. These results indicate that children with the same average lead levels but who had greater lead exposure later in childhood were at increased risk of adult criminal behavior. Specifically, a one-unit increase in the 6-year:2-year ratio (e.g., from 0.5 to 1.5) would be associated with an arrest rate 3.35 (*e*^1.21^) times greater than the arrest rate for subjects with the lower ratio, given an equal childhood average BPb. Thus, behavioral problems such as criminal activity were also more strongly associated with higher BPb concentrations at 6 years when examined using this new statistical approach.

## Discussion

Although the weight of evidence from a number of studies conducted during the last 15 years indicates that adverse effects on cognitive development occur at childhood BPb levels < 10 μg/dL, the age of greatest susceptibility has not been well defined. Several recent analyses have suggested that lead exposures at school age may be more strongly related to decrements in IQ scores or neuroanatomical deficits (Chen et al. 2005; Lanphear et al. 2005; Wasserman et al. 2000). These studies encountered problems in separating the effects at various ages due to the close tracking of BPb concentrations during childhood. Current CDC guidelines for screening children focus on 1- and 2-year olds (CDC 1991), making a better determination of the age of greatest susceptibility very important to clinical practice. The analyses presented here, for example, suggest that screening school-age children who are undergoing evaluation for cognitive deficits or behavioral problems may help to identify underlying reasons for their learning or behavioral problems.

We applied a new method that avoids the problem of serial correlation in BPb concentrations during childhood. Our method instead focused on the pattern of BPb concentrations over time by comparing the usual pattern of BPb peaking at around 2 years of age with that of children whose highest childhood exposure occurred at school age, while holding average childhood exposure constant. We found that the greatest susceptibility to both cognitive and behavioral development was at 5–6 years of age. These results suggest that several cross-sectional analyses showing stronger associations between BPb in older children and reductions in IQ scores are not simply residual effects from higher exposures at 2 years of age.

Our results are also consistent with those of Chen et al. (2005), who reported that BPb concentrations at 7 years of age had a stronger association with IQ measured at 7 years compared with BPb measured at 2 years. Our approach, however, avoided the collinearity problems of including BPb measurements at both younger and older ages in the same model.

Although the relatively large size of the combined cohorts, availability of serial BPb measurements, and numerous covariates are strengths of these analyses, there are also a few limitations. Relatively few children in these analyses had higher BPb levels at 6 years than at 2 years of age (*n* = 25, 6.3%). The model estimates, therefore, depend somewhat on the upper end of the distribution of 6-year:2-year ratios. Although relatively few children were in this upper range of the ratio, regression diagnostics did not indicate that our results were disproportionately dependent on these data. We also compared child characteristics among the 25 children with ratios > 1 with the children with lower ratios and found no significant differences for any of the covariates listed in Table 1.

Another potential limitation is that our method focuses on temporal patterns in BPb levels relative to the 2-year BPb concentration. We chose the 2-year measurement as our reference because it is typically the highest level during childhood and because it has been the focus of clinical screening efforts. Serial BPb level patterns relative to BPb measurements at ages other than 2 years may produce different results. In support of our method, however, we investigated ratios relative to ages 1 and 3 years and found similar results, although ratios using 2-year levels showed the strongest relationship to IQ and criminal arrest rates.

In conclusion, the results of this new statistical approach strengthen the emerging evidence that lead exposure in school-age children may be more strongly related to cognitive and behavioral development than exposures during earlier childhood. This finding has important implications regarding testing of children for high lead exposure. Although it is still very important to reduce lead contamination in early childhood, these analyses indicate that efforts to reduce lead exposure should continue as children progress to school age. Perhaps more directly relevant, this analysis suggests that BPb testing might be an important part of a diagnostic workup for school-age children who have cognitive delay or behavioral problems, such as attention-deficit disorder and related behavioral and learning problems. Finally, we believe that this statistical approach may be useful for disentangling the age of greatest vulnerability to other serially measured environmental toxicants such as pre- and postnatal tobacco smoke.

## Figures and Tables

**Table 1:** Characteristics of 397 children in Cincinnati and Rochester cohorts.

Characteristic	Cincinnati (*n* = 221)	Rochester (*n* = 176)	Combined (*n* = 397)
Birth weight (g)	3,144 ± 457	3225.7 ± 506	3,180 ± 480
Maternal IQ	75.2 ± 9.4	81.1 ± 12.6	77.8 ± 11.3
HOME score	32.4 ± 6.5	31.9 ± 6.3	32.3 ± 6.4
Maternal education (grade)	11.2 ± 1.4	12.2 ± 2.0	11.6 ± 1.8
Child IQ	87.0 ± 11.4	84.9 ± 14.4	86.1 ± 12.9
Child female sex	108 (48.9)	84 (47.7)	192 (48.4)
Child race (nonwhite)	197 (89.1)	125 (71.0)	322 (81.1)
BPb measures (μg/dL)			
Peak	17.9 (9.0–38.0)	9.5 (4.0–23.3)	13.6 (4.6–34.4)
Early childhood	12.0 (6.6–26.6)	5.9 (2.5–13.7)	8.9 (3.0–23.8)
Lifetime mean	11.7 (5.8–24.9)	5.8 (2.4–12.7)	8.5 (3.0–22.1)
Concurrent	7.5 (3.5–20.0)	4.2 (1.5–12.0)	6.0 (1.9–17.9)

Values are arithmetic mean ± SD, no. (%), or geometric mean (5th–95th percentile).

**Table 2:** Correlations of log of annual BPb levels (years of age).

	1	2	3	4	5	6
1	1.00	0.75	0.76	0.64	0.72	0.72
2		1.00	0.89	0.80	0.83	0.81
3			1.00	0.91	0.89	0.87
4				1.00	0.96	0.91
5					1.00	0.93
6						1.00

**Table 3:** Log-linear relationship between IQ and BPb level at each of six ages.^a^

Age (years)	β	SE	*p*-Value
1	−0.08	1.02	0.934
2	−0.46	1.08	0.670
3	−2.61	1.05	0.013
4	−2.85	1.07	0.008
5	−4.39	0.95	< 0.001
6	−3.49	1.03	< 0.001

a Adjusted for study site, birth weight, HOME score, maternal education, and IQ.

**Table 4:** Relationship of IQ to ratio of 5- and 6-year BPb to 2-year BPb (*n* = 397).

Ratio^a^	β	SE	*p*-Value
6 years:2 years	−7.00	1.54	< 0.001
5 years:2 years	−4.57	1.07	< 0.001

a Adjusted for log of average childhood BPb, site, HOME score, maternal IQ, maternal education level, and birth weight.

**Table 5:** Final model relating IQ to 6-year:2-year ratio, adjusted for site, average childhood BPb, HOME score, birth weight, maternal IQ, and maternal education level.

Variable	β	SE	*p*-Value
Ratio	−7.00	1.54	< 0.001
Ln(BPb) (childhood average)	−3.19	1.23	0.010
Study site	6.50	1.45	< 0.001
HOME score	2.23	0.81	0.007
Birth weight (g)	0.82	0.60	0.170
Maternal IQ	7.29	1.07	< 0.001
Maternal education (highest grade)	0.07	1.02	0.948

**Table 6:** Relationship of criminal arrest rate in young adults to ratio of 5- and 6-year BPb to 2-year BPb (*n* = 250).

Ratio^a^	β	SE	*p*-Value
6 years:2 years	1.21	0.36	< 0.001
5 years:2 years	1.29	0.33	< 0.001

a Adjusted for average childhood BPb, maternal IQ, socio-economic status (mean Hollingshead score), sex, and parental education level.
